# The Key Role of Peroxisomes in Follicular Growth, Oocyte Maturation, Ovulation, and Steroid Biosynthesis

**DOI:** 10.1155/2022/7982344

**Published:** 2022-02-03

**Authors:** Shan Wang, HaoXuan Yang, YongLun Fu, XiaoMing Teng, ChiChiu Wang, WenMing Xu

**Affiliations:** ^1^Department of Assisted Reproductive Medicine, Shanghai First Maternity and Infant Hospital, Tongji University School of Medicine, Shanghai, China; ^2^Joint Laboratory of Reproductive Medicine, Sichuan University-The Chinese University of Hong Kong (SCU-CUHK), West China Second University Hospital, Sichuan University, Chengdu, China; ^3^Department of Obstetrics and Gynaecology, Faculty of Medicine, The Chinese University of Hong Kong, Hong Kong, China; ^4^Reproduction and Development, Li Ka Shing Institute of Health Sciences, The Chinese University of Hong Kong, Hong Kong, China; ^5^School of Biomedical Sciences, The Chinese University of Hong Kong, Hong Kong, China; ^6^Chinese University of Hong Kong-Sichuan University Joint Laboratory in Reproductive Medicine, The Chinese University of Hong Kong, Hong Kong, China

## Abstract

The absence of peroxisomes can cause disease in the human reproductive system, including the ovaries. The available peroxisomal gene-knockout female mouse models, which exhibit pathological changes in the ovary and reduced fertility, are listed in this review. Our review article provides the first systematic presentation of peroxisomal regulation and its possible functions in the ovary. Our immunofluorescence results reveal that peroxisomes are present in all cell types in the ovary; however, peroxisomes exhibit different numerical abundances and strong heterogeneity in their protein composition among distinct ovarian cell types. The peroxisomal compartment is strongly altered during follicular development and during oocyte maturation, which suggests that peroxisomes play protective roles in oocytes against oxidative stress and lipotoxicity during ovulation and in the survival of oocytes before conception. In addition, the peroxisomal compartment is involved in steroid synthesis, and peroxisomal dysfunction leads to disorder in the sexual hormone production process. However, an understanding of the cellular and molecular mechanisms underlying these physiological and pathological processes is lacking. To date, no effective treatment for peroxisome-related disease has been developed, and only supportive methods are available. Thus, further investigation is needed to resolve peroxisome deficiency in the ovary and eventually promote female fertility.

## 1. Background

Peroxisomes, which are ubiquitous cell organelles located in a variety of tissues and organs, participate in many critical metabolic functions in humans, such as lipid metabolism, oxidative metabolic reactions, and cholesterol and plasmalogen synthesis. The ovary, an important reproductive organ, has two major functions: (1) oocyte maturation and release and (2) production of steroid hormones that are irreplaceable for folliculogenesis, menstruation cycle, sex gonad development, and maintenance of the function of the female reproductive tract [1].

Peroxisomes were first discovered by Sharma et al. in the ovary [2]. The peroxisomal enzyme catalase, which is located in the peroxisomal matrix, was discovered via 3,3′-diaminobenzidine (DAB) labelling and electron microscopic analysis of the mouse ovary. In comparison with classical peroxisomes, peroxisomes in the ovary are smaller in size and lack nucleoids; therefore, these organelles are called microperoxisomes [3]. Unlike peroxisomes in the liver, which are terminally differentiated, peroxisomes in the ovary undergo constant differentiation with each oestrus cycle [4]. Peroxisomal biogenesis disorders, which are also called diseases of the Zellweger spectrum, induce peroxisome deficiency and are characterized by very severe metabolic abnormalities that lead to a plethora of clinical symptoms. The most severe disease is Zellweger syndrome (ZS), which frequently leads to early death in childhood [5, 6]. The phenotype of children with ZS is typically characterized by severe hypotonia of the body and embryological malformations of the brain cortex, liver, kidney, and genital organs [5–10]. Regarding the reproductive system, male and female patients with ZS exhibit cryptorchism and clitoromegaly, respectively, which means that the testis might be misplaced and underdeveloped in boys and that the size of the clitoris is markedly larger in girls [11, 12]. This fact suggests that peroxisomal deficiency causes problems in genital organ development and in the oestrogen-androgen balance. However, there is little information regarding peroxisomal function in the female reproductive system, particularly in the ovary. Both the reactive oxygen species (ROS) balance and lipid metabolism are very important for oocyte maturation, ovulation, fertilization, and embryo development. Thus, our review provides the first description and summary of all the current findings related to the roles of peroxisomes in the ovary, and further research is needed for treating diseases of the female reproductive system and enhancing fertility caused by peroxisomal deletion or dysfunction.

## 2. Peroxisomal Biogenesis and Metabolic Functions

### 2.1. Peroxisomal Biogenesis

The formation of peroxisomes involves three steps, and different peroxins are needed in each step. According to the available data on peroxins, more than 30 PEX genes have been numbered, and different types of peroxins are known to be involved in different peroxisomal biogenetic processes, as follows: (1) peroxisomal membrane formation: PEX3, PEX16, and PEX19; (2) importation of peroxisomal matrix proteins: PEX5 and PEX7; and (3) fission and proliferation of peroxisomes: PEX13, PEX14, and PEX17 [13, 14].

### 2.2. Peroxisomal Metabolic Functions

Peroxisomes mainly participate in three major metabolic functions: (1) involvement in lipid metabolism; (2) maintaining the balance between ROS production and degradation; and (3) playing critical roles in phospholipid, cholesterol, and bile acid biosynthesis. Furthermore, peroxisomes are involved in retinoid and amino acid metabolism.

#### 2.2.1. Peroxisomal Fatty Acid *β*-Oxidation

Fatty acid *β*-oxidation mainly occurs in mitochondria for ATP generation, but peroxisomes are able to degrade some long-chain and very-long-chain fatty acyl-coenzyme (CoAs), 2-methyl-branched fatty acyl-CoAs, long-chain dicarboxylyl-CoAs, and the CoA esters of bile acid intermediates, which cannot be degraded in the mitochondria [15, 16]. First, fatty acids must be active in CoA derivatives and then imported into peroxisomes by peroxisomal ATP-binding cassette transporters (ABCDs) [16]. Afterwards, fatty acid *β*-oxidation occurs via four steps in peroxisomes: (1) oxidation, acyl-CoA is first desaturated to 2-trans-enoyl-CoA via a reaction catalysed by acyl-CoA oxidases (ACOX); (2) hydration, enoyl-CoA is converted to 3-hydroxyacyl-CoA; (3) dehydrogenation, the hydroxyacyl intermediate is dehydrogenated to a 3-ketoacyl-CoA; and (4) thiolytic cleavage, with the help of thiolase and sterol carrier proteins (SCPx), acetyl-CoA is released together with acyl-CoA, which is two carbon atoms shorter than the original acyl-CoA, and then enters the next round of *β*-oxidation [15–17]. Steps (2) and (3) are catalysed by a protein called multifunctional protein (MFPs), which exhibits both enoyl-CoA hydratase and 3 hydroxyacyl-CoA dehydrogenase activities [16, 18].

#### 2.2.2. Peroxisomes and ROS Metabolism

ROS include several radical species, e.g., the superoxide anion (O2·−), the hydroxyl radical (·OH), the hydroxyl radical (·OH), and hydrogen peroxide (H_2_O_2_), even though these do not possess unpaired electrons. Peroxisomes are the major location where ROS are generated and diminished [19–23]. ROS serve as a double-edged sword for cells and organs: the accumulation of oxidative damage exerts a particular toxic effect on DNA, proteins, and lipids, whereas ROS play a mediating role in a variety of important cellular processes and cell signalling pathways, such as their pivotal role in apoptosis [24, 25].

Catalase is well known as the main detoxifying enzyme that prevents the accumulation of H_2_O_2_ in cells [26, 27]. After the dismutation reaction from O_2_·− to H_2_O_2_ catalysed by SODs, H_2_O_2_ is converted into H_2_O and O_2_ [28] via a reaction catalysed by catalase [29]. The overexpression of catalase in transgenic mice leads to an extension of their life span [30], whereas the inhibition of catalase activity in the rat liver suppresses peroxisomal *β*-oxidation activity [31]. The amount of catalase is significantly reduced in tumours of the liver and other organs [32, 33]. Catalase activity is also reported to be decreased under some pathological conditions, such as ischaemia–reperfusion injury. With the exception of catalase, other enzymes located in peroxisomes, such as peroxiredoxin I and V, SOD1, glutathione S-transferase, and epoxide hydrolase, are all able to degrade ROS [23, 34].

#### 2.2.3. Peroxisome and Ether Phospholipid Synthesis

Ether phospholipid biosynthesis consists of three steps. First, glycerone phosphate is converted to acyl-glycerone phosphate (acyl-GNP) via a reaction catalysed by the peroxisomal enzyme glycerone phosphate acyl transferase (GNPAT), and acyl-GNP is then converted to alkylglycerone phosphate (alkyl-GNP) by the peroxisomal enzyme alkylglycerone phosphate synthase (AGPS) [7, 35]. The last step occurs in the endoplasmic reticulum (ER), and the reaction, which produces alkylglycerol-3-phosphate (alkyl-G-3P), is catalysed by the enzyme alkyl/acyl-GNP NAD(P)H oxidoreductase [35].

#### 2.2.4. Peroxisome and Cholesterol Synthesis

3-Hydroxy-3-methylglutaryl coenzyme A (HMG-CoA) reductase, which catalyses the rate-limiting step in cholesterol synthesis, is localized not only in the ER but also in peroxisomes [36]. Furthermore, peroxisomes contain enzymes such as isopentenyl diphosphate delta isomerase (IDI1), mevalonate kinase (MVK), phosphomevalonate kinase (PMVK), and mevalonate pyrophosphate decarboxylase (MPD), which convert mevalonate to farnesyl diphosphate (FPP) [37, 38]. Finally, peroxisomes are involved in maintaining cholesterol homeostasis [39].

## 3. Materials and Methods

### 3.1. Animals

All experimental protocols performed during this research were carried out in complete accordance to the regulatory guidelines of Animal Ethical and Welfare Committee (AEWC) of Sichuan University, China (approval code: AEWC2016, 6 January 2016). In this study, a total of 16 female C57BL6 mice were purchased from Charles River (Bei Jing, China) and keep the light for 12 h every day. After perfusion fixation, the ovaries were removed and further fixed by immersion in the same fixative overnight. The next day, complete ovaries were paraffin-embedded.

### 3.2. Immunofluorescence (IF)

For the visualization of peroxisomal proteins in the ovaries, PEX14 (1:500, Proteintech, CAT. 10594-1-AP, CHINA), catalase (1:100, Proteintech, CAT. 21260-1-AP, CHINA), and MFP2 (1:100, Invitrogen, CAT. PA582758, USA) were detected by indirect IF. The IF protocol for staining paraffin sections has been well established in our lab. Ovarian sections were first deparaffinized with xylene (3x 5 min), roasted at 60°C for 3 h and rehydrated through a series of ethanol solutions (99%, 96%, 80%, 70%, and 50% ethanol, 3 min each step). Thereafter, the sections were digested with trypsin for 9 min at 37°C to achieve better retrieval of peroxisomal antigens and improved accessibility to epitopes. The sections were then treated with 10 mM citrate buffer at pH 6.0 and heated in a microwave for 15 min at 900 W. The blocking process was performed with 4% BSA in Tris-buffered saline containing 0.05% Tween 20 (TBST) for 1 h at room temperature. After blocking, the sections were incubated with primary antibodies in 1% BSA in TBST overnight at room temperature and then with fluorochrome-conjugated secondary antibody (1:1000, Invitrogen, CAT. A11037, CHINA) for 1 h. Because the first antibody was from the same source, we used APEX™ Antibody Labelling Kits (A10468, Invitrogen, USA) to label the other same primary antibody used for double staining. The conjugated primary antibody was incubated for 14 h at 4°C, and the secondary antibody was then rinsed with TBST. Negative controls, which were incubated with TBST buffer instead of the primary antibody, were processed at the same time. The nuclei were stained with 20 *μ*l of DAPI containing antifluorescence quenching agent (P0131, 5 ml, Beyotime) for 10 min at room temperature. All pictures were obtained using a confocal laser-scanning microscope (CLSM) with an Olympus FV-1000 (40x objectives, Airy 1; averaging 16; setting pixels 2048 × 2048). The images were saved as tiff files and processed with Adobe Photoshop (Version cc 2017).

## 4. Role of Peroxisomes in the Ovary

### 4.1. Peroxisome Distribution in the Ovary

To visualize the peroxisome distribution in the ovary, we stained three representative enzymes of peroxisomes: the peroxisomal biogenesis protein PEX14 and the peroxisomal metabolic enzymes catalase and MFP2, which are two marker enzymes involved in oxidative metabolism and lipid oxidation, respectively.

**Figure 1:**
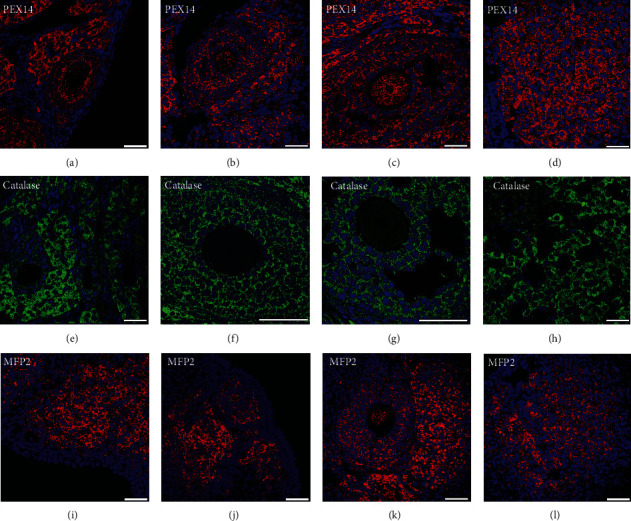
Immunofluorescence analysis of peroxisomal biogenesis proteins in developing follicles and the corpus luteum. Primary, secondary, tertiary follicles, and corpus luteus were stained with PEX14, catalase, and MFP2 antibodies and with DAPI for cell nuclei. Primary follicles are presented in (a, e, i). Secondary follicles are presented in (b, f, j). Tertiary follicles are shown in (c, g, j), and the corpus luteum is presented in (d, h, j). Scale bars = 40 *μ*m.

PEX14 is the perfect marker for visualizing peroxisomes in different cell types and organs in morphological studies [40]. Our IF results showed that peroxisomes labelled with PEX14 are numerous at all follicular stages from primordial follicles to tertiary follicles and are present in the oocyte as well as in the surrounding granulosa and theca cells (Figures 1(a)–1(d)). PEX14 revealed the highest abundance of peroxisomes in steroid hormone-producing interstitial cells followed by granulosa lutein cells of the mature corpus luteum and then granulosa and theca cells in developing follicles. This finding is in agreement with a previously published electron microscopic analysis, which revealed that interstitial cells contain more peroxisomes than granulosa cells of tertiary follicles [41]. This phenomenon has been confirmed by both catalase and MFP2 staining. High amounts of peroxisomes in gonadotropic interstitial and luteinized cells are likely needed to promote the initial steps of steroid biosynthesis and endogenous cholesterol synthesis in this cell type.

An important factor influencing oocyte maturation is the management of oxidative stress, a process in which peroxisomal catalase plays a major role. As described in Background, peroxisomes were first discovered in the ovary using its marker enzyme catalase by Böck and colleagues in 1972 [41]. Catalases were first thought to be absent in oocytes of different species [42, 43], but later, studies proved that catalase is present in oocytes at a low amount compared with other cell types of follicles [44] and is distributed in the periphery of the oocyte [45]. Our results confirm these previous findings through the use of regular concentrations (Figures 1(e)–1(h)). A longer exposure time and higher concentrations probably need to be used to visualize catalase expression in oocytes. In contrast to catalase, the other investigated peroxisomal enzymes PEX14 and MFP2 are more highly abundant in oocytes; moreover, the amount of PEX14 and MFP2 in oocytes increases during maturation from primary to tertiary follicles (Figures 1(a)–1(d) and 1(i)–1(l)).

We further found that the follicular granulosa cells surrounding oocytes contain very large amounts of catalase (Figures 1(e)–1(h)). Granulosa cells provide nutrients and signalling molecules that regulate the growth and differentiation of oocytes [46]. Communication between oocytes and granulosa cells occurs through gap junctions that are established in primary follicles [47, 48]. It is possible that oocyte-derived H_2_O_2_ is transferred through gap junctions to granulosa cells, where it is degraded by peroxisomal enzymes. ROS detoxification through gap junctions has been demonstrated in haematopoietic stem cells, which transfer ROS to the bone marrow to protect against the oxidative stress generated during haematopoietic regeneration [49].

### 4.2. Possible Functions of Peroxisomes during Follicular Development and Oocyte Maturation in the Ovary: ROS and Lipid Metabolism

#### 4.2.1. Peroxisomal Antioxidative Impacts on Ovarian Functions

Maturation of the oocyte is accompanied by increased ROS within the cells of the ovary, which might be detrimental to folliculogenesis, ovulation, and fertilization [50, 51]. Excessive ROS can lead to meiotic arrest, oocyte degeneration, development inhibition [52, 53], and zygote apoptosis [54], which leads to reduced fertility [55]. Therefore, several antioxidative enzymes, such as peroxisomal catalase, and molecular antioxidants, such as plasmalogens, function to maintain the cellular redox balance within the ovary [56, 57]. Previous researches have proved that peroxisomal abundance in the mouse oocytes is relatively constant in different oestrus cycles, but no comparison between different follicular stages was done [58]. Our IF analysis showed that the abundance of the marker protein PEX14 is significantly higher in large tertiary follicles than in primordial, primary, and secondary follicles at the same stage. With the exception of PEX14, peroxisomal MFP2 behaves similarly, which suggests coordinated and regulated proliferation of the peroxisomal compartment during the follicular phase. In contrast, the abundance of peroxisomal enzymes in the ovary during development from primary to tertiary follicles differs among different papers. Previous reports have shown that in rats and swine, the activity of catalase is particularly high in large growing and preovulatory follicles, whereas the abundance of other nonenzymatic and enzymatic ROS scavengers is reduced during follicle development [59, 60]. In contrast, another study performed in buffalos found that the concentration of catalase does not vary among differently sized follicles [61]. These discrepancies can be explained by species-, oestrus-phase-, and methodology-related differences. Behl and Pendley measured the activity of catalase in isolated granulosa cells, Parshad and Guraya measured catalase activity in isolated follicles, and Peterson and Stevenson measured the peroxisomal fraction derived from whole ovaries [59, 62, 63]. Different proteomic analyses have demonstrated the presence of catalase in follicular fluid [64] and found that its activity is higher in large follicles [60]; the reason could be that catalases are released from granulosa cells into the follicular fluid in large follicles. Interestingly, the abundance of the antioxidant enzyme family of superoxide dismutase does not vary among granulosa cells of different follicle sizes in cows [65]. In contrast, in the follicular fluid, the activity and amount of SODs are decreased in mature follicles [60, 61, 65, 66], and this decrease may be explained by the demand for ROS in ovulation. In preovulatory follicles, ROS production is upregulated in parallel with cytokines, prostaglandins, proteolytic enzymes, and steroids, which leads to blood flow alterations and eventually to follicle rupture [67]. This process is similar to an acute inflammatory reaction because many inflammatory genes and reagents are induced by the LH surge during this ovulation process [68–70]. In summary, an appropriate ROS concentration is fundamental for providing an appropriate environment for ensuring physiological activation during the oestrus cycle.

In common to the above-discussed studies and in accordance with our theory of the protective role of peroxisomes within ovarian cells, regulation of the oxidative stress level is of key importance during folliculogenesis. Several experiments have shown that catalase is essential for ROS scavenging in the ovary. For example, catalase protects oocyte DNA from oxidative damage during meiosis [71], prevents apoptosis of the oocyte and follicles [62, 72], prevents regression of the corpus luteus during pregnancy [73], contributes to follicle selection [62], and reduces ovary damage during periods of hibernation [74]. In addition to catalase, the peroxisomal enzyme GNPAT plays an indirect role in the control of the oxidative stress levels in cells. Plasmalogens are phospholipids that can be found in any cell as components of the plasma and organellar membranes and are highly susceptible to oxidative stress. Plasmalogens influence membrane dynamics and are particularly abundant in oocytes [75, 76]. In recent years, plasmalogens have been proposed to function as endogenous antioxidants in ROS trapping and in the protection of cells against the damage resulting from lipid peroxidation [35, 77]. In accordance with the higher requirement for oxidative stress defence at the later stages of oocyte maturation [59, 62, 63], GNPAT was found to be the most highly abundant in the oocytes of tertiary follicles and is particularly localized in large peroxisomes in the periphery of the oocyte [78]. Taken together, these results strongly indicate that peroxisomes are needed for successful progression through the follicular phase and for the scavenging of ROS during folliculogenesis.

#### 4.2.2. Peroxisomal Lipid *β*-Oxidation Regulation of Ovarian Functions

In oocytes, nutritional lipids are stored as lipid droplets, which undergo changes in their number, composition, size, and aggregation during follicle maturation [79, 80]. GNPAT is involved in the synthesis of triacylglycerols and other nonether glycerolipids, which are important nutritional lipids [81]. Similar to GNPAT, MFP2, a peroxisomal fatty acid *β*-oxidation enzyme [82], and ABCD3 [83], a lipid transporter involved in peroxisomal lipid metabolism displays higher abundance in oocytes of tertiary follicles, which suggests an involvement of peroxisomal lipid metabolism in follicular development of the oocyte during the late stages of folliculogenesis [58]. The mechanism through which the metabolism of lipids is regulated and influences oocyte development remains under investigation but has not been fully clarified. The functional relationship of lipid droplets and mitochondria has been previously postulated due to their close proximity [84]. In oocytes, fatty acids stored within lipid droplets are transferred to mitochondria for ATP generation through *β*-oxidation, a process that is increased during oocyte maturation and at the preovulatory phase [85]. In follicles, the nutritional supply and metabolism of lipids provide an important source of ATP for oocyte growth, regulate the fluidity and integrity of the oocyte membrane, determine the viability and developmental competence of oocytes, and influence the composition of the follicular fluid and the efficiency of fertilization [84, 86]. The inhibition of *β*-oxidation has repercussions on acquisition of the developmental competence of the oocyte and fertility by blocking the resumption of meiosis and interfering with nuclear maturation and embryonic development [85, 87]. Nevertheless, most of the available information regarding *β*-oxidation in the ovary is related to mitochondrial *β*-oxidation, and the function of peroxisomal *β*-oxidation in this organ remains to be elucidated. Although peroxisomal *β*-oxidation is not directly involved in cellular ATP provision, it shortens long-chain and very-long-chain fatty acids for further metabolization within mitochondria. Furthermore, peroxisomal *β*-oxidation degrades a broad spectrum of (toxic) lipid intermediates that cannot be processed by mitochondria, as presented in Background. Therefore, peroxisomes might be responsible for regulation of the homeostasis of fatty acid species such as the long-chain fatty acid linoleic, oleic, stearic, and palmitic acids, which are detrimental for oocyte development [88]. As discussed in the former chapter, ovulation is accompanied by increased production of prostaglandins and cytokines [89] and activation of proteolytic enzymes such as matrix metalloproteinase [90]. Interestingly, peroxisomal *β*-oxidation is of major importance for the in vivo chain shortening of prostaglandins [91].

**Figure 2:**
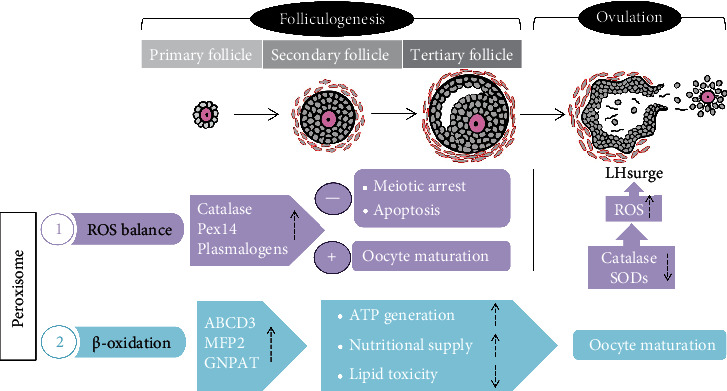
Schematic representation of peroxisomal regulation during folliculogenesis. Folliculogenesis is initiated with primordial follicles, which develop into primary follicles, secondary follicles, tertiary follicles, and finally preovulatory follicles. Under an LH surge triggered by oestrogen positive feedback, ovulation occurs for fertilization. This consecutive and synchronized event is accompanied by peroxisomal regulation to maintain the ROS balance and ensure the *β*-oxidation reaction. The peroxisomal marker enzyme catalase and peroxisomal biogenesis factor are induced during follicular maturation, whereas SODs are downregulated. A suitable concentration of ROS during different stages of the follicular phase is fundamental for ensuring oocyte development and ovulation. In addition to antioxidation, the peroxisomal *β*-oxidative enzymes ABCD3 and MFP2 are induced during follicular maturation and provide sufficient ATP and nutrition for oocyte growth, the selection of dominant oocytes, and the degradation of excessive lipid toxicity to maintain the efficiency of fertilization. The up arrow means “upregulation” while the down arrow stands for “downregulation.”

The general regulation of peroxisomes during follicular development is illustrated in Figure 2. However, the molecular mechanisms underlying follicular development have not been fully elucidated, and additional evidence related to the regulation of peroxisomes during this process is needed.

### 4.3. Possible Function of Peroxisomes in the Corpus Luteum

The corpus luteum is a very important endocrine structure in female ovaries because it can produce a large amount of progesterone and moderate amounts of oestrogen and inhibin A. Moreover, the corpus luteum contributes to maintenance of the early period of pregnancy. When the ovum is not fertilized, the corpus luteum degenerates into fibrous scar tissue called the corpus albicans. In 1972, Böck first described the peroxisome distribution in the mouse ovarian corpus luteum, and the highest level of peroxisomes in the corpus luteum has been found in pregnant females, as demonstrated by the finding that the corpus luteum exhibited stronger staining than interstitial cells and markedly stronger staining than granulosa cells of tertiary follicles. In addition, the peroxisome size is slightly larger (0.2–0.3 *μ*m) in granulosa lutein cells than in granulosa cells of tertiary follicles (0.2 *μ*m), and the peroxisomal profile is markedly less frequent in granulosa cells of tertiary follicles than in granulosa lutein cells [41]. In contrast to granulosa cells in developing follicles, peroxisomes show a very heterogeneous distribution in distinct individual luteinized granulosa cells. This strong heterogeneity was also noted by Böck through catalase staining of the corpus luteum [41]. According to his results, peroxisomes are situated within fields of the smooth endoplasmic reticulum (ER), in the vicinity of lipid droplets and close to mitochondria, which could reflect the organelle interaction and transfer of metabolic intermediators during endogenous cholesterol synthesis and steroid synthesis [41]. The literature indicates that granulosa cells are the specific cell type for oestrogen synthesis because aromatase is only localized in this cell type [92]. Interestingly, aromatase is also very heterogeneously located in the macaque corpus luteum, and the strongly stained cells are interspersed among other weakly stained cells [93]. Accordingly, the heterogeneous peroxisome distribution pattern may reflect different levels of active steroidogenesis in these cells. During steroidogenesis, a large amount of ROS is generated. Therefore, it is not surprising that the peroxisomal antioxidative enzyme catalase is highly abundant in large granulosa lutein cells, as will be discussed in detail in a later section.

### 4.4. Regulatory Mechanisms of Peroxisomal Genes in the Ovary

The correct progression through the oestrus cycle is timed by the concerted action of gonadotropic hormones, including follicle-stimulating hormone (FSH), luteinizing hormone (LH), and human chorionic gonadotropin (hCG), which are produced in the pituitary gland and placenta. An increasing body of evidence shows that these hormones influence and are influenced by peroxisome proliferator-activated receptors (PPARs) [94]. PPARs are ligand-activated receptors that regulate disparate metabolic processes, such as lipid and glucose metabolism and cell and organellar proliferation and differentiation [95, 96]. In particular, these receptors are linked to the proliferation of peroxisomes and the modulation of their ROS and lipid metabolism in various organs [97, 98]. We speculate that the function of peroxisomes in the ovary is hormonally regulated through the transcription factor class of PPARs.

All three PPARs (PPAR*α*, *β*, and *γ*) have been detected in the ovaries of different species [99]. In contrast to PPAR*α* and PPAR*β*, which are constantly present throughout the ovarian cycle and folliculogenesis [99, 100], the expression of PPAR*γ* increases in preovulatory follicles and markedly decreases after the LH surge (after hCG) [99, 101]. PPAR*γ*, which is the most intensively studied PPAR in the ovary, is detected primarily in granulosa cells and is suggested to influence their function during follicular maturation as well as their intercellular communication with oocytes [102]. The inhibition of PPAR*γ* severely affects the development of oocytes [102]. In 2000, Viergutz et al. indicated that PPAR*γ* plays a role in arresting the cell cycle in lutein cells to maintain their differentiated state [103]. Due to loss of PPAR*γ*, one in three females become sterile, and the remaining females become subfertile [104]. These effects may be due to insufficient ovarian function, and this insufficient ovarian function is related to the capability of the corpus luteum to produce sufficient progesterone to support the establishment of pregnancy [102]. Indeed, it has been reported that PPARs are involved in the regulation of 17*β*-oestradiol and progesterone release by the porcine corpus luteum [105].

### 4.5. Roles of Peroxisomes in Ovarian Steroid Synthesis

#### 4.5.1. Link between Peroxisomal *β*-Oxidation and Steroid Synthesis

The first hint that linked peroxisomes with steroid synthesis is the localization of some proteins involved in the steroidogenic pathway in peroxisomes. Steroid synthesis is a complex metabolic process that utilises cholesterol as the obligatory precursor [106]. Cholesterol is either synthesized de novo from acetate via a complex process involving almost 30 different enzymes or obtained from the diet. Interestingly, the presqualene segment of the cholesterol biogenetic pathway is localized in peroxisomes [38, 107–109]. In addition, the key rate-limiting enzyme in cholesterol synthesis, HMG-CoA reductase, is an enzymatic component of peroxisomes [36, 110]. A mouse model has further proven that peroxisomal plays indispensable roles in cholesterol formation. It has been reported that the cholesterol biogenetic pathway and overall cholesterol regulation are disturbed in the liver of PEX2-knockout mice [37]. The above-described evidence has linked peroxisomes with steroid synthesis through the indispensable precursor “cholesterol.”

Moreover, some direct evidence describes the role of peroxisomal enzymes in steroid synthesis. Sterol carrier protein 2 (SCP2) is a peroxisomal *β*-oxidation protein involved in peroxisomal *β*-oxidation of branched-chain fatty acids and bile acid formation from cholesterol. This protein harbours 3-ketoacyl-CoA thiolase activity in the N-terminal domain and SCP2 in the C-terminal domain [8, 111, 112] and is enriched in hormone-producing cells and gonads [113–115]. Interestingly, SCP2 serves as the conductor in the cytoplasm for transporting cholesterol into the outer mitochondrial membrane, and after this step, cholesterol is imported into mitochondria via the StAR protein for further steroid synthesis [116–121]. Further evidence has shown that SCP2 is highly expressed in the steroidogenic compartments of the rat ovary and is upregulated after gonadotropin stimulation at both the mRNA and protein levels [122, 123]. Additionally, the peroxisomal enzyme MFP2, also called 17-*β*-hydroxysteroid dehydrogenase 4 (17*β*-HSD4), catalyses the hydration and dehydrogenation process during *β*-oxidation [15, 16]. This enzyme is reportedly involved in the oxidative step of steroid conversion from oestradiol to estrone. 17*β*-HSD4 is an 80 kDa protein with an N-terminally cleaved enzymatically active fragment of 32 kDa that is capable of conducting the dehydrogenase step not only with *β*-oxidation but also with steroids at the C17 position. This was the first proven enzyme that exhibits dehydrogenase activity with not only 3-hydroxyacyl-CoA derivates of fatty acids but also steroids [124, 125].

The above-described evidence shows the subcellular location of enzymes involved in steroidogenesis and indicates that peroxisomes are directly linked to the hormone production process. To date, very little evidence has been found regarding peroxisomal molecular regulation in ovarian steroidogenesis. In contrast, the regulation of peroxisomes in steroidogenic endocrine regulation has been documented in the testes and adrenal gonads of male animals. In the male adrenal cortex, the number of peroxisomes is upregulated after inhibition of the conversion of cholesterol to pregnenolone [126]. Similarly, the blockage of HMG-CoA reductase with mevilonin or downregulation of the cholesterol levels with nafenopin in serum results in the proliferation of peroxisomes in male rat adrenal gonads [127, 128]. More interestingly, testosterone secretion from rat Leydig cells was observed in parallel with the peroxisome levels by Mendis-Handagama and colleagues [129].

#### 4.5.2. Peroxisomal ROS Production and Antioxidant Regulation during Steroid Synthesis

Many studies have investigated the peroxisomal enzyme catalase in steroidogenic regulation. Catalase shows different distributions among ovarian cycles, and catalase activity is significantly improved in different mammals after gonadotropin stimulation [62, 130–132]. Behl and Pandey further proved that the enhancement of catalase activity parallels oestradiol upregulation after FSH stimulation. Moreover, the degree of the increase was greater in large follicles than in medium and small follicles [62]. FSH promotes follicular development and the selection of dominant follicles [133]; during this stage, oestradiol reaches its highest concentration, and the catalase content increases consistently with the increase in oestradiol after FSH stimulation, which suggests a role for catalase in the selection of dominant follicles and the prevention of atresia. Moreover, catalase activity is positively correlated with the amount of cytochrome P450scc and ferredoxin, which are two components of the steroidogenic electron transport chain in both rat and pig ovaries [134]. In the steroidogenic metabolic pathway, a large amount of ROS is produced [135–137] and then converted via a reaction catalysed by SODs to H_2_O_2_ [29, 138] for further detoxification by glutathione peroxidase or catalase. At the midluteal phase, the SOD1 levels are greatly increased after hCG stimulation [139]. Because SOD1 is localized not only in the cytoplasm but also in peroxisomes [140], the extent to which peroxisome-derived SOD1 contributes to the degradation of these oxidative radicals is unclear. After its production, H_2_O_2_ is further degraded by catalase. Thus, catalase, as an ROS cleaner, may act as a protective factor to maintain the balance of ROS concentrations during steroid synthesis and maintain the physiological reaction of hormone synthesis.

#### 4.5.3. Underlying Mechanisms for Steroidogenic Disorder under Peroxisomal Deficiency

The roles of peroxisomes in steroid synthesis were proven in granulosa cell lines by Baumgart-Vogt's laboratory [78], and hormone secretion and steroidogenic proteins are detected after PEX13 knockdown in granulosa tumour cell lines—KK1 cells [141]. PEX13 knockdown can induce whole peroxisomal dysfunction by blocking peroxisomal matrix protein import. In vitro studies have revealed that pregnenolone and progesterone synthesis are strongly downregulated when peroxisomes are dysfunctional, and this finding might be explained by the strong reduction of StAR protein after PEX13 knockdown. Similarly, the oestradiol levels are strongly downregulated, which suggests an alteration of 17*β*-hydroxysteroid dehydrogenase activity. Additionally, ROS production is enhanced after PEX13 knockdown in KK-1 cells.

**Figure 3:**
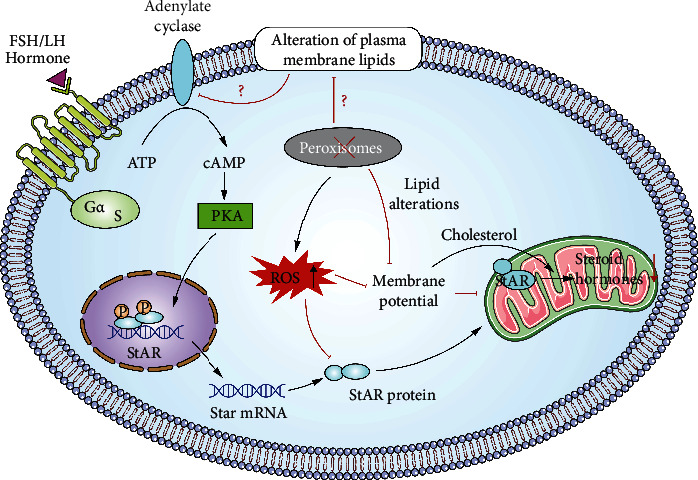
Possible mechanism of peroxisomal regulation during steroidogenesis. According to the literature, the steroidogenic pathway is disturbed under peroxisomal-deficient conditions. The underlying mechanism may at least partly involve inhibition of the rate-limiting enzyme StAR. Excessive oxidative stress caused by peroxisomal deficiency can directly lead to the inhibition of steroid synthesis; moreover, the mitochondrial membrane potential is disturbed under peroxisomal-deficient conditions, which deteriorates StAR-mediated cholesterol transport. The specific molecular mechanism of StAR inhibition needs to be clarified in the future. Additionally, the lipid structure and lipid composition of the cell membrane are closely related to the activity of adenylyl cyclase (AC) and G protein-coupled receptor signalling. We hypothesize that peroxisomes may be involved in upstream cell signalling for hormone production by regulating the membrane composition of steroidogenic cells. Red line stands for “inhibit” while black line means “promote.”

ROS are formed during aerobic metabolism, and the physiological content of ROS is important for cellular activities such as cell signalling and ovulation [142–144]. However, excessive amounts of ROS can cause toxic effects on normal ovarian functions and fertility disorders [55, 145]. ROS cleaners, such as SODs, catalase, and glutathione peroxidase (GPX) [146], are increased under peroxisome-deficient conditions, which indicates that peroxisomal deficiency can induce a relative antioxidative response [78]. A similar phenotype has also been observed in Sertoli cell-specific PEX13-knockout mice [147]. Excessive ROS exert negative impacts on hormone synthesis by blocking cholesterol transport into mitochondria in male Leydig cells [148] and female luteal cells [138]. In addition, H_2_O_2_ can inhibit adenylyl cyclase activity and reduce progesterone production [149, 150]. This finding was explained by Diemer and colleagues and is due to dissipation of the mitochondrial membrane potential [151], which is critical for StAR-mediated cholesterol translocation [152]. Interestingly, peroxisomal dysfunction can lead to mitochondrial dysfunction. PEX5-knockout mice exhibit very severe mitochondrial abnormalities in the steroid-producing adrenal cortex [153], and complex III of the mitochondrial respiratory chain is strongly reduced in Sertoli cell-specific PEX13-knockout mice [147]. Two reasons most likely explain the mitochondrial dysfunction observed under peroxisome-disturbed conditions: (1) excessive ROS production induces direct toxic effects and (2) accumulated toxic fatty acids that cannot be degraded by peroxisomes overload the degradation ability of mitochondria. Last but not least, peroxisomal disturbance leads to reduced StAR protein expression, as described above, and the identity of the transcription factor of StAR or the posttranscriptional step that is influenced is worth investigating. The possible mechanism of peroxisomal regulation during steroidogenesis is shown in Figure 3.

## 5. Human Disease Caused by Peroxisomal Deficiency

As described in Background, peroxisomes are indispensable for normal cellular function; therefore, peroxisome dysfunction can lead to serious biochemical abnormalities and result in various clinical symptoms, diseases, and even death. To date, two types of peroxisomal disorders have been identified: peroxisome biogenesis disorders (PBDs) and single peroxisomal metabolic enzyme-related disorders [154]. The biogenesis of peroxisomal membrane protein and matrix protein relies on peroxin (Pex) genes, and mutations in any of the related genes can lead to PBDs [5, 13]. Well-known PBDs are divided into two groups: ZS disorders (ZSDs) and rhizomelic chondrodysplasia puncta (RCDPs) [37]. ZS is the most severe and well-known disease caused by peroxisomal deficiency, and its manifestation involves severe hypotonia of the body and embryological malformations in the kidney, liver, and central nervous system. ZS can also lead to adrenal cortex degeneration and consequently adrenal deficiency [5–10]. Less severe ZSDs include neonatal adrenoleukodystrophy (NALD) and infantile Refsum disease (IRD) [5, 6]. Compared with individuals with ZS, patients with NALD and IRD display less severe clinical manifestations. Patients with NALD can survive for more than ten years, and many patients with IRD are expected to survive up to their third decade [155]. Patients with RCDP usually demonstrate skeletal abnormalities, and the underlying cause of RCDP is either mutations of enzymes involved in plasmalogen biosynthesis or problems with peroxisomal targeting signal- (PTS-) 2-specific protein import [9, 155].

## 6. Peroxisome-Related Dysfunction in the Reproductive System

ZS is associated with clitoromegaly in girls or cryptorchism in boys, which indicates that peroxisome dysfunction can cause problems in the development of genital organs and an imbalance in androgen-oestrogen secretion [11]. Many of the lines of evidence obtained to date are related to peroxisomal dysfunction in male fertility. Studies have proven that peroxisome metabolism is critical for sexual organs and that peroxisomal dysfunction can result in adrenocortical insufficiency, spermatogenesis defects, and even complete testicular degeneration [11, 12]. In addition to clinical manifestations, many mouse models have described the negative influence of peroxisomal gene knockout on male reproductive health. For example, ACOX1-deficient mice exhibit reduced amounts of Leydig cells and spermatids [156]. In addition, arrest of spermatogenesis and atrophic testes have been observed in GNPAT-knockout mice [157]. Moreover, the knockout of MFP2 causes fatty acid accumulation in Sertoli cells and seminiferous tubules, an incomplete germinal epithelium and a reduction in elongated spermatids, which results in male infertility [158]. Baumgart-Vogt used a Sertoli cell-specific Pex13-knockout mouse model to investigate peroxisomal function in the testis and found that the mouse model exhibited “Sertoli cell only” syndrome (SCO) [147]; additionally, strong accumulation of peroxisome-metabolized fatty acids (VLCFAs, phytanic acid, and pristanic acid) and neutral lipids are observed in the testis, and large intratubular vacuoles are observed in seminiferous tubules [147]. All of these findings are consistent with testicular disorders often found in patients with ZS and/or X-ALD/AMN.

Compared with peroxisomal insufficiency-related pathologies related to male reproductive health, almost no studies have investigated the pathological alterations in the female ovary. According to the literature, only two papers describe peroxisomal gene-knockout female mice. Rodemer et al. found a reduced ovary size in GNPAT-knockout mice, and the number of secondary and tertiary follicles and corpora lutea was reduced, but no influence was observed on intact follicles [157]. Another study found that female ACOX-knockout mice present smaller ovaries and infertility [159]. However, these two papers only describe the phenotypes; the underlying molecular mechanism remains to be clarified in the future.

## 7. Possible Therapy against Peroxisome-Related Disease

Peroxisomal deficiency-caused ZSDs exhibit very severe symptoms in the neonatal period and later in adolescence or adulthood and frequently leads to early death in childhood. At present, no curative therapy has been developed, and only supportive care is available. Through peroxisomal *β*-oxidation, tetracosahexaenoic acid is converted to docosahexaenoic acid (DHA), a long-chain polyunsaturated fatty acid that is important for retinal and brain function [160, 161]. Patients with ZSDs have low levels of DHA in membranes of erythrocytes, and DHA supplementation may be helpful for these patients, but this finding remains debated [162–164]. Lorenzo's oil leads to lower VLCFAs in plasma and may helpful for patients with X-ALD [165–167] but is useless for disease progression [168, 169]. Plasmalogens are critical for cell membrane formation, indeed patients with ZSD, that usually have low amount of plasmalogens, present problems in cell membrane formation [170, 171]. It has been proven that supplementation with precursors of plasmalogens (batyl alcohol) can increase the erythrocyte plasmalogen levels and improve clinical symptoms in some patients [172–174]. In addition, some ongoing clinical trials have identified several compounds that can stimulate peroxisomal biogenesis and function in vitro [175–179]. Hopefully, these compounds will be administered to patients to improve peroxisomal function in the future. Moreover, gene therapy has been introduced but still needs to be verified in human trials [180, 181]. Orthotopic liver transplantation has been applied to one 6-month-old patient with ZSD, and hepatocyte transplantation was administered to another 4-year-old patient [182, 183]. This therapy is helpful for decreasing the concentrations of VLCFAs and pipecolic acid and improves the bile acid profiles in these patients. Unfortunately, the effects of peroxisomal therapy on ovarian functions have not been investigated. According to our review, supplementation with precursors of plasmalogens may contribute to improving oocyte quality by ensuring membrane function and trapping ROS.

As described in the literature, only treatments against oxidative conditions derived from peroxisomal deficiency have been developed. Antioxidant supplementation can reduce oxidative stress caused by excessive ROS in humans [55]. Both enzymatic and nonenzymatic antioxidants have been proven to be helpful for the removal of excessive ROS [55]. It has been reported that antioxidant supplementation is beneficial for the oocyte quality in mouse [184]. In addition, the antioxidant melatonin, a very important naturally produced antioxidant in mammals, is able to prevent the ROS-mediated deterioration of the oocyte quality in rats [185, 186] and humans [187, 188]. In addition to melatonin, resveratrol can improve the number and quality of oocytes of mice and protect against the reduction of fertility observed with reproductive ageing [189], and it has also been proved that resveratrol plays a protective role against premature ovarian failure (POF) and prompts female germline stem cell survival in mice [190]. Jiang et al. have shown that the possible mechanism through which ROS induces POF involves the suppression of telomerase reverse transcriptase (TERT) activity [191]. Nonenzymatic antioxidants such as vitamins A, C [189], and E [192, 193] are reportedly able to reduce the oxidative stress marker in women with endometriosis but have no effects on the fertilization rate [193]. N-acetyl-cysteine (NAC) is able to increase the intracellular GSH concentrations and/or directly scavenge free radicals [194, 195]. After the administration of a combination of NAC and folic acid, patients with unexplained recurrent pregnancy loss achieve better outcomes [196]. More studies are needed to investigate the effects of antioxidant supplementation as a possible treatment therapy for these patients. The daily intake of fresh green vegetables, fruits, antioxidant-rich legumes, and plant products that contain high levels of antioxidants may be beneficial for reducing ROS [197]. The administration of additional antioxidants to neutralize excessive ROS production caused by peroxisomal deficiency may be an effective therapy in the future because the ROS balance is critical for follicular maturation, oocyte development, and mitochondria function involving hormone production.

## 8. Conclusion

Due to the increase in the infertility rate observed in recent years, researchers have shown growing interest in investigating the regulation of ovarian function and oocyte maturation. The deterioration of oocyte quality will lead to an increased probability of aneuploidy and miscarriage. The redox balance is closely related to female subfertility and infertility. With the exception of ROS and antioxidants, lipid regulation and metabolism also play indispensable roles on oocyte maturation, ovulation, fertilization, and embryo development. In this review, we thoroughly present the expression and regulation of some major peroxisomal antioxidants and *β*-oxidative enzymes during follicular development, oocyte maturation, ovulation, corpus luteum function, and steroidogenesis. This review provides the first systematic analysis of the role of peroxisomes in ovarian functions. However, the molecular mechanism underlying these roles has not been fully investigated, and further studies are needed to elucidate the specific regulatory pathway through which peroxisomes affect ovarian function, which would provide further insights for the treatment of female infertility in vivo.

## Additional Points

*Summary Sentence*. Peroxisomes are strongly involved in ovarian follicular growth, oocyte maturation, ovulation, and steroid biosynthesis to maintain normal fertilization
